# Comparative Fecal Microbiota Analysis of Infants With Acute Bronchiolitis Caused or Not Caused by Respiratory Syncytial Virus

**DOI:** 10.3389/fcimb.2022.815715

**Published:** 2022-03-07

**Authors:** Flavio De Maio, Danilo Buonsenso, Delia Mercedes Bianco, Martina Giaimo, Bruno Fosso, Francesca Romana Monzo, Michela Sali, Brunella Posteraro, Piero Valentini, Maurizio Sanguinetti

**Affiliations:** ^1^ Dipartimento di Scienze di Laboratorio e Infettivologiche, Fondazione Policlinico Universitario A. Gemelli IRCCS, Rome, Italy; ^2^ Dipartimento di Scienze Biotecnologiche di Base, Cliniche Intensivologiche e Perioperatorie, Università Cattolica del Sacro Cuore, Rome, Italy; ^3^ Dipartimento di Scienze della Salute della Donna, del Bambino e di Sanità Pubblica, Fondazione Policlinico Universitario A. Gemelli IRCCS, Rome, Italy; ^4^ Dipartimento di Scienze della Vita e Sanità Pubblica, Università Cattolica del Sacro Cuore, Rome, Italy; ^5^ Istituto di Biomembrane, Bioenergetica e Biotecnologie Molecolari (IBIOM), Consiglio Nazionale delle Ricerche, Bari, Italy; ^6^ Dipartimento di Scienze Mediche e Chirurgiche, Fondazione Policlinico Universitario A. Gemelli IRCCS, Rome, Italy

**Keywords:** acute bronchiolitis, infants, fecal microbiota, respiratory syncytial virus, respiratory effort, severe illness

## Abstract

Bronchiolitis due to respiratory syncytial virus (RSV) or non-RSV agents is a health-menacing lower respiratory tract (LRT) disease of infants. Whereas RSV causes more severe disease than other viral agents may, genus-dominant fecal microbiota profiles have been identified in US hospitalized infants with bronchiolitis. We investigated the fecal microbiota composition of infants admitted to an Italian hospital with acute RSV (25/37 [67.6%]; group I) or non-RSV (12/37 [32.4%]; group II) bronchiolitis, and the relationship of fecal microbiota characteristics with the clinical characteristics of infants. Group I and group II infants differed significantly (24/25 [96.0%] versus 5/12 [41.7%]; *P* = 0.001) regarding 90% oxygen saturation (SpO_2_), which is an increased respiratory effort hallmark. Accordingly, impaired feeding in infants from group I was significantly more frequent than in infants from group II (19/25 [76.0%] versus 4/12 [33.3%]; *P* = 0.04). Conversely, the median (IQR) length of stay was not significantly different between the two groups (seven [3–14] for group I versus five [5–10] for group II; *P* = 0.11). The 16S ribosomal RNA V3–V4 region amplification of infants’ fecal samples resulted in 299 annotated amplicon sequence variants. Based on alpha- and beta-diversity microbiota downstream analyses, group I and group II infants had similar bacterial communities in their samples. Additionally, comparing infants having <90% SpO_2_ (*n* = 29) with infants having ≥90% SpO_2_ (*n* = 8) showed that well-known dominant genera (*Bacteroides*, *Bifidobacterium*, *Escherichia*/*Shigella*, and *Enterobacter*/*Veillonella*) were differently, but not significantly (*P* = 0.44, *P* = 0.71, *P* = 0.98, and *P* = 0.41, respectively) abundant between the two subgroups. Overall, we showed that, regardless of RSV or non-RSV bronchiolitis etiology, no fecal microbiota-composing bacteria could be associated with the severity of acute bronchiolitis in infants. Larger and longitudinally conducted studies will be necessary to confirm these findings.

## Introduction

Bronchiolitis is a disease of the lower respiratory tract of infants and young children that, despite being characterized by small airway obstruction, can progress to pneumonia, respiratory failure, apnea, or death (Meissner, 2016). Based on recently published data, bronchiolitis accounted for 313,566 emergency department visits between 2006 and 2010 in the United States (4.3% involved children were <2 years of age) (Hasegawa et al., 2014) and for 3.4 million hospital admissions in 2005 worldwide (Nair et al., 2010). According to the American Academy of Pediatrics (AAP), a viral upper respiratory prodromal phase heralds increased respiratory effort and wheezing, which are hallmarks of lower respiratory tract illness (Ralston et al., 2014). As an etiological agent of bronchiolitis (Florin et al., 2017), respiratory syncytial virus (RSV) was the most frequently detected (50–80%), followed by rhinovirus or parainfluenza virus (5–25%) and by influenza virus (1–5%), in children from North America hospitals (Meissner, 2016). Global estimates by the World Health Organization in 2014 indicate that RSV accounted for more than 80% of lower respiratory tract infections in infants younger than 1 year (Piedimonte and Perez, 2014). Among local, single-center, *ad-hoc* studies so far conducted on RSV infections in children in Italy (Azzari et al., 2021), one study showed that 103 (16.5%) of 624 RSV-related hospitalizations over a 5-year period (September 2014–August 2019) required admission to the intensive care unit (Barbati et al., 2020). Of these, 70.9% (73/103) and 45.6% (47/103) involved children <3 months and <1 month of age, respectively.

Management of viral bronchiolitis largely relies on supportive care (supplemental oxygen combined with hydration remains the mainstay) (Florin et al., 2017), whereas RSV prophylaxis is limited to high-risk infants (Piedimonte and Perez, 2014). Although it is still unknown if RSV (alone or with other co-infecting viruses) may cause more severe disease than rhinovirus or other viruses (Biagi et al., 2020), previous evidence is that bronchiolitis cases due to rhinovirus may result in a length of hospital stay (LOS) shorter than in cases due to RSV (Meissner, 2016). Nonetheless, severe illness, defined as a longer LOS, a greater requirement for intensive care, and a higher risk of apnea, remains undesirable (Meissner, 2016).

The pathophysiology of bronchiolitis suggests the existence of distinct underlying mechanisms that, in turn, generate syndrome subtypes with overlapping clinical symptoms (e.g., wheezing) but with heterogeneous treatment responses (Polack et al., 2019). In two multicenter studies of children <2 years of age admitted to hospital with severe bronchiolitis, four distinct clinical profiles were identified using a hypothesis-free statistical clustering approach (Dumas et al., 2016). Two of them (A and B) differed markedly in terms of wheezing history (more frequent in profile A) and viral etiology (more rhinovirus infection in profile A versus more RSV infection in profile B). Concomitantly, in a multicenter case-control study of hospitalized infants <12 months of age with bronchiolitis, four distinct fecal microbiota profiles were identified using an unbiased clustering approach (Hasegawa et al., 2016). In particular, the proportion of infants was significantly lowest in the *Enterobacter*/*Veillonella*-dominant profile and highest in the *Bacteroides*-dominant profile.

The aim of the present study was to perform a comparative fecal microbiota analysis according to the RSV (group I) or non-RSV (group II) infection status of infants less than 6 months of age who were hospitalized with acute bronchiolitis in Italy. In the two infants’ groups, fecal microbiota characteristics were evaluated in relation to the characteristics that define the clinical course of bronchiolitis.

## Materials and Methods

### Study Design, Setting, and Subjects

In this study, we explored the fecal microbiota composition and its relationship with the characteristics of bronchiolitis in infants who were infected or not infected by RSV. To this end, we enrolled infants aged <6 months among those (*n* = 49) who were admitted to our hospital (Fondazione Policlinico Universitario A. Gemelli IRCCS, Rome, Italy) with a diagnosis of acute bronchiolitis within 3 months (October through December) of the 2019 winter season. According to AAP guidelines (Ralston et al., 2014), the attending physician used a combination of rhinitis, cough, tachypnea, wheezes/crackles (rales), and retractions to make diagnosis. Infants (*n =* 12) who had diarrhea (or other gastrointestinal illness symptoms) or had received antibiotics within 7 days were excluded. After enrollment, viral etiology of bronchiolitis was determined on infants’ nasopharyngeal aspirates using a polymerase chain reaction (PCR)-based assay (ePlex system; GenMark Diagnostics, Carlsbad, CA, USA). This assay was able to detect 18 respiratory viruses (adenovirus; coronaviruses 229E, HKU1, NL-63, and OC43; human metapneumovirus; human rhinovirus/enterovirus; influenza viruses A, A/H1, A/H1-2009, and A/H3; influenza virus B; parainfluenza viruses 1, 2, 3, and 4; RSV A and RSV B). Based on positive or negative PCR results, infants were classified as having RSV (25 infants; 67.6%) or non-RSV (12 infants; 32.4%) bronchiolitis, respectively (**Table 1**). Infants who tested negative for RSV were found to be positive for rhinoviruses (6 infants), influenza viruses (2 infants), or none of PCR-assay detectable viruses (4 infants). Four of 25 infants who tested positive for RSV were also found to be positive for rhinoviruses but were only included in the RSV bronchiolitis group. We collected infants’ demographics, medical history, and acute illness information through a standardized structured parents’ interview. Of 37 infants, 20 (13 with RSV bronchiolitis and 7 with non-RSV bronchiolitis) received systemic antibiotics after fecal sampling (**Table 1**).

**Table 1:** Characteristics of 37 infants with RSV or non-RSV bronchiolitis as assessed by nasopharyngeal secretion PCR testing.

Characteristics	RSV bronchiolitis group (*n* = 25)	Non-RSV bronchiolitis group (*n* = 12)	*P*-value
Demographics			
Age, months, mean (SD)	2.16 (0.44)	3.83 (0.88)	0.05
Male sex	17 (68.0)	6 (50.0)	0.24
Caucasian race	23 (92.0)	12 (100)	0.45
Birth history			
Cesarean delivery	11 (44.0)	7 (58.3)	0.32
Prematurity (32–37 weeks)	4 (16.0)	2 (16.7)	0.65
ED presentation signs/symptoms			
Fever	8 (32.0)	7 (58.3)	0.12
Dyspnea	24 (96.0)	7 (58.3)	0.001
Rhinitis	13 (52.0)	5 (41.7)	0.41
Increased respiratory effort indices^a^			
Retractions	20 (80.0)	7 (58.3)	0.0001
Wheezes/Crackles (rales)	22 (88.0)	7 (58.3)	0.0001
Tachypnea	10 (40.0)	3 (25.0)	0.0001
Oxygen saturation (SpO_2_) <90%	24 (96.0)	5 (41.7)	0.001
Impaired feeding	19 (76.0)	4 (33.3)	0.04
(Co)infection by viruses other than RSV^b^			
Rhinovirus	4 (16.0)	6 (50.0)	0.021
Influenza virus	0 (0.0)	2 (16.7)	0.026
Systemic antibiotic use after bronchiolitis diagnosis^c^	13 (52.0)	7 (58.3)	0.49
ICU and intubation			
No ICU	21 (84.0)	12 (100)	0.19
ICU without intubation	2 (8.0)	0 (0.0)	—
ICU with intubation	2 (8.0)	0 (0.0)	—
LOS, days, median (IQR)	7 (3–14)	5 (5–10)	0.11

Data are no. (%) of infants unless otherwise indicated. —, not computed.

ED, emergency department; ICU, intensive care unit; IQR, interquartile range; LOS, length of hospital stay; PCR, polymerase chain reaction; RSV, respiratory syncytial virus; SD, standard deviation; SpO_2_, saturation of peripheral oxygen.

a Of listed indices, SpO_2_ <90% value for each infant was determined through repeated measurements by pulse oximetry.

b According to PCR testing performed on the nasopharyngeal secretions used to determine the RSV or non-RSV etiology of bronchiolitis.

c Diagnosis was concomitant with the fecal sampling that was, indeed, obtained before patients were treated with antibiotics (see text for details).

The Ethics Committee of our Institution approved the study (approval number 0013018/20), and written informed consent was obtained from a parent of all infants included in the study.

### Fecal Sample Collection and 16S Ribosomal RNA Gene Sequencing

At the time of hospitalization (within 24 h from admission), fecal samples were obtained from infants’ diapers and then placed in sterile feces collection containers, which were immediately stored at −80°C until microbiota analysis.

For each sample, DNA extraction was performed in a strictly controlled level-2 biological safety workplace. In keeping with a previously described protocol (Posteraro et al., 2019), we suspended 200-mg fecal sample in hexadecyltrimethylammonium bromide (CTAB) buffer, and this suspension was used to extract bacterial DNA with the DANAGENE MICROBIOME Fecal DNA kit (Danagen-Bioted, Barcelona, Spain). The extracted DNA was quantified using a Qubit 4 fluorometer (Thermo Fisher Scientific, Waltham, MA, USA), and then subjected to the 16S ribosomal RNA (rRNA) V3–V4 region amplification using adapter-containing V3_Next_For (5’-TCGTCGGCAGCGTCAGATGTGTATAAGAGACAGCCTACGGGNGGCWGCAG-3’) and V4_Next_Rev (5’- TCTCGTGGGCTCGGAGATGTGTATAAGAGACAGGACTACHVGGGTATCTAATCC-3’) primers (Klindworth et al., 2013). The resulting amplicons were purified using Agencourt AMPure XP beads (Beckman Coulter, Indianapolis, IN, USA), and then barcoded using the Nextera XT Index kit (Illumina, San Diego, CA, USA). The indexed amplicons were diluted at an equimolar ratio and then pooled to prepare a sequence library *via* the 2 × 300-bp paired-end protocol in the MiSeq instrument (Illumina). The PhiX v3 internal control (Illumina) was added to increase base diversity in the sequence library.

### Bioinformatics and Statistics Analyses

After demultiplexing, FastQ forward and reverse sequence reads were analyzed using the QIIME2 (v.2020.6) microbiome analysis pipeline (Bolyen et al., 2019). Briefly, trimming of merged reads allowed removing Illumina adapters and non-biological primer sequences. Next, reads were subjected to quality filtering and chimera removal to generate amplicon sequence variants (ASVs) using the DADA2 algorithm (Callahan et al., 2016). We used both the pre-fitted sklearn-based taxonomy classifier (https://docs.qiime2.org/2021.8/plugins/available/feature-classifier/classify-sklearn/) and SILVA 132 database (https://www.arb-silva.de) for taxonomic ASVs’ annotation. Pre-processing of final data allowed to remove both mitochondrial sequences and less than 0.01% represented microbial taxa (Karstens et al., 2019).

We used R 4.0.2 (https://www.rstudio.com/) and phyloseq (McMurdie and Holmes, 2013) statistical packages for downstream analyses of alpha (e.g., inverse Simpson) and beta (e.g., Bray–Curtis distance) microbial community’s diversity. Before that, we rarefied each sample to a depth of 70,000 reads to restrict uneven sampling effects. Differences between infants’ groups according to alpha diversity metrics were assessed using the Mann–Whitney *U*-test, whereas those according to Bray–Curtis (http://www.econ.upf.edu/~michael/stanford/maeb5.pdf) or weighted UniFrac (Lozupone et al., 2007) distance matrix-computed beta diversity metrics were assessed using the permutational multivariate analysis of variance (PERMANOVA).

Additionally, differences in clinical characteristics between infants’ groups were assessed using the Mann–Whitney *U*-test for continuous variables or the Chi-square test for categorical variables.

In all analyses, statistical significance was set at a *P <*0.05.

## Results

### Description of RSV or Non-RSV Bronchiolitis Infants

**Table 1** summarizes the characteristics of 37 infants admitted to our hospital for an acute bronchiolitis due to RSV (group I, 25 infants) or non-RSV (group II, 12 infants). Seventeen (68.0%) of 25 infants and 6 (50.0%) of 12 infants were male, with 11 (44.0%) and seven (58.3%) infants being born by Cesarean delivery, respectively. The mean age of group I infants was 2.16 months (standard deviation [SD], 0.44), which differed (albeit at the statistical significance limit) from the mean age of group II infants (3.83 months; SD, 0.88; *P* = 0.05).

Before hospitalization, infants had presented to the emergency department with severe illness symptoms such as dyspnea (24/25 [96.0%] versus 7/12 [58.3%]) or oxygen saturation <90% (24/25 [96.0%] versus 5/12 [41.7%]), which differed significantly between group I and group II infants (*P* = 0.001, for both comparisons). Nineteen of 24 infants with increased respiratory effort had impaired feeding, which accounted for 76.0% (19/25) of group I infants. This proportion was significantly higher than that of group II infants (33.3% [4/12]; *P* = 0.04).

During hospitalization, all (100%) of 12 infants from group II did not require intensive care unit (ICU) admission compared to 21 (84.0%) of 25 infants from group I (two of four ICU admitted infants were intubated), but this difference did not reach statistical significance (*P* = 0.19). Similarly, no statistically significant difference was observed regarding the median LOS (with interquartile range [IQR]) between infants’ groups (5 [5–10] for group II versus 7 [3–14] for group I; *P* = 0.11).

### Fecal Microbiota Characterization in RSV or Non-RSV Bronchiolitis Infants

The 16S rRNA gene sequencing of fecal samples from 37 infants included in the study resulted in 4,370,836 reads, accounting for 1,529 annotated ASVs. Rarefaction curves showed enough sequencing depth to allow all samples to be biologically explained (**Figure S1**). Accordingly, each sample’s plateau reaching curve suggested sufficient reads to describe the bacterial community from each infant’s feces. Finally, further data processing allowed us to obtain 4,182,668 reads, which accounted for 299 annotated ASVs.

**Figure 1:**
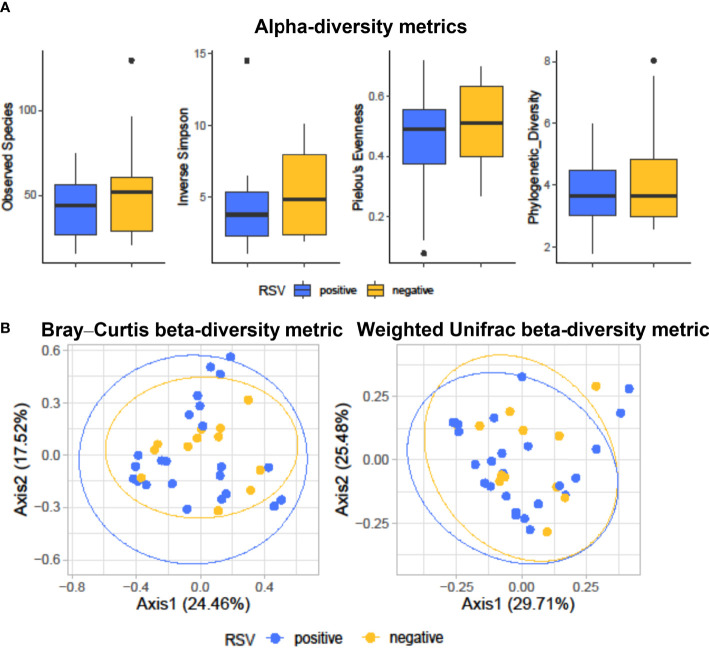
Alpha-diversity **(A)** and beta-diversity **(B)** analyses of fecal bacterial communities from hospitalized infants with acute bronchiolitis. In **(A)**, species richness, equitability, and phylogenetic diversity (mean ± standard deviation) values were compared between RSV-positive (blue-colored) and RSV-negative (yellow-colored) infants’ groups, respectively. In each boxplot, outliers are shown. Except for the phylogenetic diversity index that shows no difference, the observed species, inverse Simpson, and Pielou’s evenness indices are slightly (but not significantly) lower in RSV-positive than in RSV-negative infants. In **(B)**, Bray–Curtis or weighted UniFrac compositional-based distances were computed for RSV-positive (blue-colored) and RSV-negative (yellow-colored) infants’ groups, respectively. The principal coordinate analysis (PCoA) results are presented as two-dimensional ordination plots, which were generated using two (axis1 and axis2) principal coordinates. These results show no significant separation between RSV-positive and RSV-negative infants’ groups. In both **(A, B)**, “positive” and “negative” indicate infants whose nasopharyngeal samples were, respectively, positive for RSV or negative for RSV at molecular testing (see text for details). RSV, respiratory syncytial virus.

**Figure 1A** shows diversity and evenness of fecal bacterial communities from RSV-positive (group I, *n* = 25) and RSV-negative (group II, *n* = 12) infants with bronchiolitis. Four alpha-diversity metrics, such as observed species index, inverse Simpson index, Pielou’s evenness, and phylogenetic diversity, were assessed (in terms of mean ± SD) and compared between groups. RSV-positive infants’ bacterial communities had lower species diversity (observed species, 41.4 ± 16.0; inverse Simpson, 4.0 ± 2.8) than those in RSV-negative infants (observed species, 55.8 ± 31.4; inverse Simpson, 5.2 ± 2.9; *P* = 0.18 for both comparisons). Conversely, equitability or phylogenetic diversity assessments indicated similarity between RSV-positive and RSV-negative infants’ bacterial communities with respect to species predominance (Pielou’s evenness, 0.4 ± 0.2 and 0.5 ± 0.1, respectively; *P* = 0.24) or phylogenetic distance (3.9 ± 1.4 and 3.9 ± 1.4, respectively; *P* = 0.51).

**Figure 1B** shows the principal coordinate analysis (PCoA) of Bray–Curtis- or weighted UniFrac-matrix distances depicting the fecal microbiota composition for RSV-positive and RSV-negative groups of infants with bronchiolitis. Consistent with previous findings (Hasegawa et al., 2016), analyses showed different compositional profiles that, however, overlapped between infants’ groups. PERMANOVA analyses definitively showed that the microbiota of RSV-positive infants did not significantly separate from that of RSV-negative infants (Bray–Curtis distance-based analysis, *P* = 0.68; weighted UniFrac distance-based analysis, *P* = 0.87).

**Figure 2:**
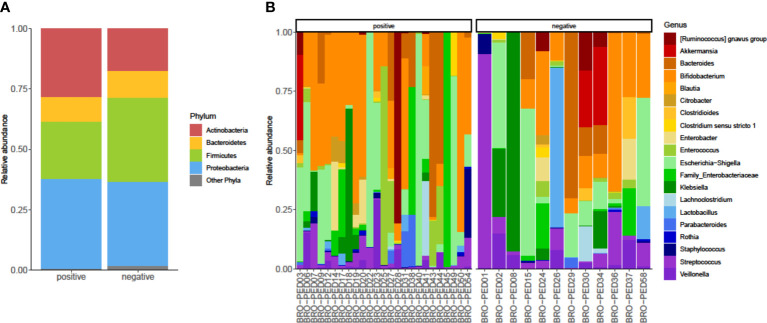
Relative abundances of bacterial taxa composing the fecal bacterial communities from hospitalized infants with acute bronchiolitis. For RSV-positive and RSV-negative infants’ groups, the proportions of major bacterial phyla **(A)** and top-20 bacterial genera **(B)** were computed, normalized, and presented as stacked-bar plots per group of **(A)** or single **(B)** fecal samples (here indicated with designation codes, e.g., bronchiolitis (BRO)-pediatrics (PED)03), respectively. Shown is the phyla *Actinobacteria*, *Bacteroidetes*, *Firmicutes*, and *Proteobacteria* along with other phyla, which include *Fusobacteria*, *Tenericutes*, and *Verrucomicrobia*. In each plot **(A)** or group of plots **(B)**, “positive” and “negative” indicate infants whose nasopharyngeal samples were, respectively, positive for RSV or negative for RSV at molecular testing (see text for details). RSV, respiratory syncytial virus.

As shown in **Figure 2A**, we identified bacterial phyla with higher (*Actinobacteria*, *Bacteroidetes*, *Firmicutes*, and *Proteobacteria*) or lower (*Fusobacteria*, *Tenericutes*, and *Verrucomicrobia*) relative abundances in the infants’ fecal microbiota. *Actinobacteria* and *Firmicutes* displayed a relative, albeit not significant, opposite trend between RSV-positive and RSV-negative infants’ groups. *Actinobacteria* and *Firmicutes* abundances were 17% and 28%, respectively, in RSV-positive infants and were 23% and 33%, respectively, in RSV-negative infants. In both infants’ groups, *Bacteroidetes* and *Proteobacteria* abundances were 10% and 35%, respectively. At the bacterial genus level (**Figure 2B**), *Escherichia*/*Shigella* (5% and 3%, respectively), *Bifidobacterium* (7% and 5%, respectively), and *Bacteroides* (6% and 5%, respectively) were most relatively abundant in both RSV-positive and RSV-negative infants. This was consistent with the fecal microbiota dominance by genera (i.e., *Escherichia*, *Bifidobacterium*, and *Bacteroides*) previously identified in bronchiolitis infants (Hasegawa et al., 2016).

### Relationship Between Fecal Microbiota and Bronchiolitis Characteristics

We performed subgroup analyses of infants who presented (29 infants, of which 24 were RSV positive) or not presented (eight infants, of which seven were RSV negative) with oxygen saturation (SpO_2_) <90% (**Table 1**), which was here used as an increased respiratory effort index. We found that *Bacteroides* and *Bifidobacterium* were relatively more abundant in the 29 infants’ subgroup (7% and 5%, respectively) than in the eight infants’ subgroup (6% and 2%, respectively), but these differences were not statistically significant (*P* = 0.44 and *P* = 0.71, respectively). Likewise, genera such as *Escherichia*/*Shigella* and *Enterobacter*/*Veillonella* were relatively more, but not significantly (*P* = 0.98 and *P* = 0.41, respectively), or equally abundant in the 29 infants’ subgroup (6% and 6%, respectively) than in the eight infants’ subgroup (4% and 6%, respectively).

## Discussion

Our fecal microbiota investigation of hospitalized infants with acute bronchiolitis did not show any statistically significant difference in their bacterial community related to the RSV or non-RSV etiology. Clinically, infants who tested positive for RSV had significantly more severe illness than infants who tested negative for RSV, thus supporting previous findings from Italian children studies (Fabbiani et al., 2009; Midulla et al., 2010). Regardless of viral etiology, severely ill infants had a bacterial community more markedly but not significantly populated by certain genera than less severely ill infants. One genus was *Bacteroides*, and this finding was consistent with the observed higher likelihood of bronchiolitis in infants with a *Bacteroides*-dominant profile than in infants with an *Enterobacter*/*Veillonella*-dominant profile of fecal microbiota (Hasegawa et al., 2016). Another genus was *Bifidobacterium*, and this finding was consistent with the observed equal likelihood of bronchiolitis for infants with a *Bifidobacterium*-dominant profile and those with an *Enterobacter*/*Veillonella*-dominant profile of fecal microbiota (Hasegawa et al., 2016). Taken together, these findings suggest the lack of significant association between fecal genera such as *Bacteroides* or *Bifidobacterium* and the extent or severity of the bronchiolitis course.

While both humoral and cytotoxic immune response arms are required to control RSV infection, especially in infants (Griffiths et al., 2017), gut microbiota-produced metabolites, such as short-chain fatty acids (SCFAs), can modulate immune cell functions both systemically and locally (Machado et al., 2021). Thus, no surprise is that low or even null SCFAs’ concentrations in the feces correlated with greater susceptibility to respiratory viral infection (Sencio et al., 2021). It is also intuitive that immunomodulatory SCFAs’ functions increase the viral pathogen clearance and, meanwhile, decrease the exacerbated inflammation-driven tissue damage in the lungs (Machado et al., 2021). However, as much of the viral load is cleared by the time RSV infection manifests clinically (Piedimonte and Perez, 2014), it was reasonable for us to hypothesize an inflammatory response-mitigating fecal microbiota potentially able to affect the bronchiolitis severity or duration in our infants. In this context, it should be recalled that the gut microbiota-modulating probiotic (e.g., *Lactobacillus acidophilus* NCFM) consumption was able to reduce the incidence and duration of cold- or influenza-like symptoms in children (Leyer et al., 2009). Otherwise, it was reasonable for us to hypothesize that infants who needed supplemental oxygen and had impaired feeding might have an altered fecal microbiota owing to a more severe illness.

We studied two (RSV or non-RSV) bronchiolitis infants’ groups that were at least homogenous regarding variables such as sex, race, stress (i.e., Cesarean delivery), prematurity history, or antibiotic use (i.e., assessed before fecal sampling), which are well known to alter the gut microbial community profile (Sommer and Bäckhed, 2013). While alpha- and beta-diversity microbiota analyses showed both infants’ groups to share similar bacterial communities in their feces, however, as above mentioned, microbiota analyses in infants’ subgroups allowed us to note marked but not statistically significant differences in terms of relative abundance of bacterial populations from relevant phyla or, more specifically, genera. Among usually adopted criteria to evaluate bronchiolitis severity in infants and young children (i.e., oxygen therapy or ventilatory support requirement, LOS, and (pediatric) ICU admission) (Biagi et al., 2020), we focused on the less than 90% SpO_2_, which is a severe hypoxemia-indicating parameter that reflects unequivocally the need for supplemental oxygen. It was then conceivable (albeit not provable) that naturally *Bacteroides*- or *Bifidobacterium*-enriched feces in our infants could drive an acute respiratory distress syndrome (e.g., documented by means of increased respiratory effort indices) or, in some instances, an evolving respiratory failure that required ventilatory assistance. Considering that a rapid assortment of microbial communities occurs in the first months of life, we cannot rule out a potential confounding effect of the age difference (albeit not statistically significant) between the two groups of infants on the results of our microbiota study. Otherwise, we weighed our study groups for variables known to be associated with the risk of developing severe disease (Piedimonte and Perez, 2014). For example, prematurity in our infants (four with RSV bronchiolitis and two with non-RSV bronchiolitis) might have implied the immaturity to control ventilation as well as the partial missing of the third trimester window, during which the transfer of maternal IgG to the fetus occurs or the maturation of T cells culminates (Piedimonte and Perez, 2014).

Despite its monocentric and observational nature, this study attempts, for the first time to our knowledge, to elucidate the relationship of the fecal microbiota composition with the RSV or non-RSV bronchiolitis characteristics in hospitalized infants. One strength of this study was to include a sizeable number of infants (37 in total) 3-month aged on average. This makes our study’s findings comparable to those from another clinically important study published on the topic that is, however, representative of a non-Italian infants’ context. In that study (Hasegawa et al., 2016), 40 infants (median age, 3 months) hospitalized with bronchiolitis were characterized with respect to their fecal microbiota. It is remarkable to find our infants’ microbiota characteristics be like those of infants studied by Hasegawa et al. (2016). Consistently, this likeness reinforces the finding of certain bacterial genera (e.g., *Bacteroides*) potentially affecting the bronchiolitis course in our infants. The present study’s results are also reminiscent of those from a very recent investigation that showed an altered gut microbiota to be associated with RSV disease severity (Harding et al., 2020). However, restricting our study to a single sample from each enrolled infant hindered us from providing any statistical inference or causality of the fecal bacterial composition with the illness severity as well as from investigating the differences in bacterial composition at the species level or in bacterial functionality between bronchiolitis infants’ groups. Nonetheless, our study can be the starting point for larger and longitudinally conducted future studies. Microbiota patterns that will emerge from these studies may be the input of data mining approaches aimed to discover specific bronchiolitis phenotypes to which target therapeutic treatments.

In conclusion, we showed no statistically significant variation in the fecal microbiota composition of hospitalized infants according to the RSV or non-RSV etiology of bronchiolitis. Additionally, we showed that fecal microbiota-composing bacteria could not be significantly associated with the acute bronchiolitis clinical course in infants. Thus, our findings weaken the significance of gut microbiota modulation-based strategies as key interventions to prevent and control the complications of this health-menacing lower respiratory tract disease.

## Data Availability Statement

The datasets presented in this study can be found in online repositories. The names of the repository/repositories and accession number(s) can be found below: National Center for Biotechnology Information (NCBI) BioProject database under accession number PRJNA781954.

## Ethics Statement

The studies involving human participants were reviewed and approved by the Fondazione Policlinico Universitario A. Gemelli IRCCS Ethics Committee. Written informed consent to participate in this study was provided by the participants’ legal guardian/next of kin.

## Author Contributions

FDM and MG performed the experiments. DMB, FRM, and MiS helped perform the experiments. FDM, DB, MG, and BF analyzed the data. MiS, BP, PV, and MaS conceived the study and supervised the study conduction and the data analysis. BP wrote the paper. FDM, DB, and MaS helped write the paper. All the authors read and approved the final version of the manuscript.

## Conflict of Interest

The authors declare that the research was conducted in the absence of any commercial or financial relationships that could be construed as a potential conflict of interest.

## Publisher’s Note

All claims expressed in this article are solely those of the authors and do not necessarily represent those of their affiliated organizations, or those of the publisher, the editors and the reviewers. Any product that may be evaluated in this article, or claim that may be made by its manufacturer, is not guaranteed or endorsed by the publisher.

## Supplementary Material

Supplementary Figure 1 Rarefaction curves obtained from 37 fecal samples of RSV or non-RSV bronchiolitis infants subjected to microbiota characterization. Sequencing depth is shown for each sample based on the number of observed species. RSV, respiratory syncytial virus.
