# Supplementary figures and images for: Comparative Fecal Microbiota Analysis of Infants With Acute Bronchiolitis Caused or Not Caused by Respiratory Syncytial Virus
Source: Front Cell Infect Microbiol. 2022 Mar 7;12:815715. doi: 10.3389/fcimb.2022.815715 (PMC8940166; doi:10.3389/fcimb.2022.815715)

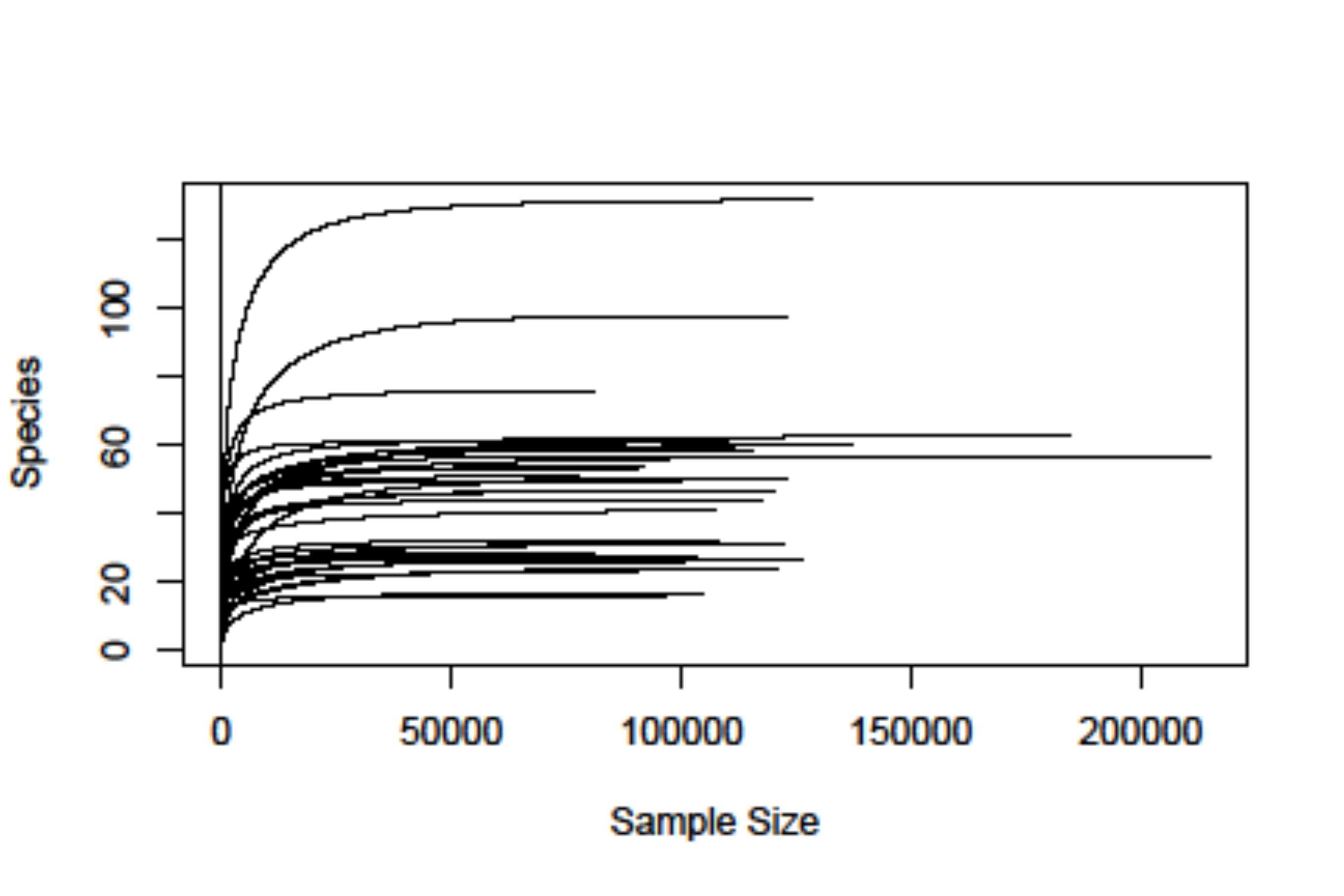

Supplement: Supplementary Figure 1 — Rarefaction curves obtained from 37 fecal samples of RSV or non-RSV bronchiolitis infants subjected to microbiota characterization. Sequencing depth is shown for each sample based on the number of observed species. RSV, respiratory syncytial virus. [file Image_1.tif]
